# QT-interval evaluation in primary percutaneous coronary intervention of ST-segment elevation myocardial infarction for prediction of myocardial salvage index

**DOI:** 10.1371/journal.pone.0192220

**Published:** 2018-02-08

**Authors:** Andrea Igoren Guaricci, Patrizia Carità, Valentina Lorenzoni, GraziaPia Casavecchia, Mark Rabbat, Riccardo Ieva, Natale Daniele Brunetti, Daniele Andreini, Matteo Di Biase, Giancarlo Marenzi, Antonio Bartorelli, Mauro Pepi, Gianluca Pontone

**Affiliations:** 1 Institute of Cardiovascular Disease, Department of Emergency and Organ Transplantation, University Hospital “Policlinico” of Bari, Bari, Italy; 2 Department of Medical and Surgical Sciences, University of Foggia, Foggia, Italy; 3 Department of Cardiology, University Hospital P. Giaccone, Palermo, Italy; 4 Scuola Superiore Sant'Anna, Firenze, Italy; 5 Loyola University of Chicago, Chicago, Illinois, United States of America; 6 Edward Hines Jr. VA Hospital, Hines, Illinois, United States of America; 7 Centro Cardiologico Monzino, IRCCS, Milan, Italy; 8 Department of Cardiovascular Sciences and Community Health, University of Milan, Milan, Italy; 9 Department of Biomedical and Clinical Sciences “Luigi Sacco”, University of Milan, Milan, Italy; University of Messina, ITALY

## Abstract

Assessing the efficacy of revascularization therapy in patients with ST-segment elevation myocardial infarction (STEMI) is extremely important in order to guide subsequent management and assess prognosis. We aimed to determine the relationship between corrected QT-interval (QTc) changes on standard sequential ECG and myocardial salvage index in anterior STEMI patients after successful primary percutaneous coronary intervention. Fifty anterior STEMI patients treated by primary percutaneous coronary intervention underwent quantitative ECG analysis and cardiac magnetic resonance. For each patient the difference (ΔQTc) between the QTc of ischemic myocardium (maximum QTc in anterior leads) versus remote myocardium (minimum QTc in inferior leads) during the first six days after STEMI was measured. The QTc in anterior leads was significantly longer than QTc in inferior leads (p<0.0001). At multivariate analysis, ΔQT_C_ and peak troponin I were the only independent predictors for late gadolium enhancement while ΔQTc and left ventricular ejection fraction were independent predictors of myocardial salvage index <60%. The receiver operative curve of ΔQTc showed an area under the curve of 0.77 to predict a myocardial salvage index <0.6. In conclusion, in a subset of patients with a first occurrence of early revascularized anterior STEMI, ΔQTc is inversely correlated with CMR-derived myocardial salvage index and may represent a useful parameter for assessing efficacy of reperfusion therapy.

## Introduction

Assessing the efficacy of revascularization therapy in patients with anterior ST-segment elevation myocardial infarction (STEMI) is extremely important in order to guide subsequent management and assess prognosis. Estimation of infarct size with late gadolinium enhancement (LGE) technique and assessment of myocardial salvage index (MSI) measured by cardiac magnetic resonance (CMR) within the first week after primary percutaneous coronary intervention (pPCI) are independently related to early ST-segment resolution, adverse LV remodelling [1,2], major adverse cardiac events (MACE) and mortality at mid-term follow-up [3,4]. However, the limited availability and access to CMR creates a need for simpler methods for patient prognostication.

Typically, significant changes of ventricular repolarization are detected early by standard 12-lead-ECG in patients with STEMI [5,6]. Whether changes of corrected-QT-interval (QTc) after STEMI reperfusion reflect a modification of ischemic myocardial tissue is unknown.

Thus, we sought to determine the relationship between QTc changes on standard sequential ECG and MSI in anterior-STEMI patients after successful pPCI.

## Methods

### Study population

From a cohort of 208 consecutive patients with STEMI referred to the Centro Cardiologico Monzino in Milan between January 2011 and June 2015, we identified 86 patients with anterior-STEMI. The diagnosis was based on symptoms, ECG findings (ST-segment elevation) and proximal or middle left anterior descending artery (LAD) lesions were included. Exclusion criteria were: a) previous MI (8 patients); b) atrial fibrillation or use of antiarrhythmic drugs (6 patients); c) catecholamine administration (2 patients) d) PCI performed >12h after chest pain onset (4 patients); e) bundle branch block (3 patients); f) incomplete reperfusion (2 patients); g) electrolyte imbalance (2 patients); h) conduction disorders such as atrio-ventricular-block (1 patient) and intermittent pre-excitation (1 patient); i) technical difficulties in QT measurement (4 patients); j) pericardial effusion (1 patient); and k) enrolment in STEMAMI Study (active treatment) (2 patients). Therefore, 50 patients with a first anterior-STEMI were finally included in the study. The study was approved by the university ethics review board (Comitato Etico degli IRCCS Istituto Europeo di Oncologia e Centro Cardiologico Monzino). All patients signed informed consent and the study conformed to the ethical guidelines of the 1975 Declaration of Helsinki

### Electrocardiogram collection and analysis

For each patient, standard 12-lead-ECGs (paper speed of 25mm/s, standardization of 10mm/1 mV) were recorded at admission, within the first hour after pPCI, and every 24h for the first six hospital days. For each ECG, the QT was measured in all leads from the onset of the QRS to the end of the T-wave on the isoelectric baseline [7]. The isoelectric baseline was defined by the reference line between two PQ intervals. The end of the T-wave was defined as the return to the isoelectric baseline. When the U-wave followed the T-wave, QT was measured to the nadir of the curve between the T and U-waves. The measurement was made for each anterior (V2-V5) and inferior lead (II, III and aVF). The QTc was obtained using Bazett’s formula: QTc = QT/√RR [8].

For each patient, the ΔQTc-AI-MA defined as the maximum dispersion between anterior and inferior leads (maximum anterior QTc—minimum inferior QTc) and the ΔQTc-AI-ME defined as the difference between the mean QTc values in anterior and inferior leads were calculated. Electrocardiographic data were evaluated twice by an expert reader (with ≥5 years of clinical experience) blinded to patient clinical history and data. Another expert reader repeated ECG data assessment. Kappa values were calculated for inter-observer and intra-observer variability.

### Cardiac magnetic resonance protocol

All patients were studied with a 1.5T scanner (Discovery MR450; GE Healthcare, Milwaukee, WI) after pPCI. After acquiring localizer images of the heart, breath-hold steady-state-free-precession cine sequences were obtained using the following parameters: echo time 1.57msec, 15 segments, repetition time 46msec without view sharing, slice thickness 8mm, field of view 350mmx263 mm, and pixel size 1.4mmx2.2 mm. A cine short axis stack, 2-, 3-, and 4-chamber long axis views of the left ventricle (LV) were acquired [9]. Breath-hold T2-weighted short-TI inversion-recovery fast-spin-echo pulse sequence was performed for the quantification of area at risk (AAR). The LV was entirely encompassed by contiguous slices in short-axis orientation. Each slice was obtained during an end-expiratory breath hold of 12 to 15s, depending on the patient's heart rate. The sequence parameters were FOV: 380to400mm; TR: two R-R intervals, TE: 100msec, TI: 150msec, matrix: 256x192, slice thickness: 8mm. Then, 0.1mmol/kg of Gadolinium-BOPTA (Multihance, Bracco, Milan, Italy) was administered at a flow rate of 3mL/s followed by a 20mL saline flush. Ten minutes after contrast injection, breath-hold contrast-enhanced segmented T1-weighted inversion-recovery gradient-echo sequences were acquired with the same prescriptions for cine images to detect late gadolinium enhancement (LGE).

### Image interpretation

CMR data were transferred to a dedicated workstation and analysed with a commercially available cardiac software (Report Card 4.0; GE Healthcare, Milwaukee, WI). Using T2-weighted short-axis images, infarct-related edema was considered present when the signal intensity (SI) of the myocardium was >2SD the mean SI of the contralateral remote region of normal myocardium [2]. The hyper-intense regions were tracked in order to outline the extent of AAR, measured in grams and expressed as a percentage of LV. On LGE imaging, MI was considered present if the SI of hyper-enhanced myocardium was >5SD the mean SI of the remote region [10]. Microvascular obstruction was defined as a hypo-enhanced region within infarcted myocardium. MI sizing was similarly obtained by manually tracking the regions of interest and measured in grams and percent of LV mass. MSI was calculated using the following equation [(AAR—infarct size at LGE)/AAR] [11]. CMR data was evaluated twice by an expert reader (with ≥5 years of clinical experience in CMR performance and analysis) blinded to patient clinical history and data. Another expert reader repeated CMR data evaluation. Kappa values were calculated for inter-observer and intra-observer variability.

### Statistical analysis

Continuous variables are expressed as mean±SD or median [27–75 percentile], whereas categorical variables are expressed as percentages. During hospital stay, QTc changes were evaluated with paired Student T-test. Spearman’s rank correlation coefficient (r) was used to evaluate correlation between ΔQTc-AI-MA at different time points and CMR findings. Linear regression was used to study the relationship between log-transformed LGE and ΔQTc, considering demographic and clinical variables as potential confounders. Multivariable models were developed including variables with p<0.1 at univariate analysis. The association between MSI and ΔQTc was similarly analysed with logistic regression analysis. A receiver-operator characteristic (ROC) curve was performed to evaluate the ability of ΔQTc to discriminate patients with MSI <0.6 vs. those with MSI >0.6. The Youden method was used to find the best cut-off. Statistical analysis was performed with STATA version 12.1 (StataCorp., College Station, Texas) and R version 3.0.2. A p value <0.05 was considered statistically significant.

## Results

The baseline clinical, echocardiographic and laboratory data of the fifty patients (44 males, mean age 59±10 years) with a first anterior STEMI enrolled in the study are listed in Table 1. All patients underwent successful pPCI and optimal medical therapy. The AAR was (35[25–58] g, 37±22% of the LV myocardial mass) and LGE was (19[7–29] g, 16[6–26]% of the LV myocardial mass) with an average MSI of 0.45±32% (Table 2). The inter-observer agreement between the two readers for MSI was 0.93 with a kappa value of 0.85 (95% confidence interval [CI] 0.60–1.0). Intra-observer agreement for MSI of the two readers was 0.91 with a kappa value of 0.76 (95% CI 0.42–1.0) and 0.95 with a kappa value of 0.85 (95% CI 0.60 to 1.0), respectively. The QTc increased in all patients (admission QTc 435±39 msec vs. peak QTc 506±57 msec; p<0.001).

**Table 1:** Baseline characteristics of patient population.

Characteristics	
Age (years)	59±10
Male, n(%)	44(88)
*Cardiovascular risk factors*, *n(%)*	
Hypertension	19(38)
Diabetes	4(8)
Hypercholesterolemia	21(42)
Family history of CAD	22(44)
Current smoker	29(58)
Killip class I	49(98)
*Laboratory parameters*	
Creatinine at admission (mg/dl)	0.93±0.23
eGFR at admission (ml/min/1.73mq)	93±25
Kalemia at admission (mEq/l)	3.86±0.45
Peak kalemia (mEq/l)	4.35±0.37
Peak troponin I (ng/mL)	50[18–112]
*Medication at hospital admission*	
Beta-blockers	4(8.0)
ACEi/ARBs	13(26.0)
Diuretics	3(6.0)
Ca-antagonist	3(6.0)
Anticoagulants agents	3(6.0)
Nitrate	1(2.0)
Tricagrelor	13(26.0)
Prasugrel	30(60.0)
Clopidogrel	7(14.0)
Statins	3(6.0)
Aspirin	48(96.0)
Gp IIb/IIIa inhibitors	9(18.0)
*Catheterization laboratory data*	
Time-to-PCI (min)	171[100–180]
Door-to-balloon time (min)	37[0–45]
*Infarct-related artery*, *n(%)*	
Proximal LAD	26(52.0)
Single-vessel disease	31(62.0)
Double-vessel disease	14(28.0)
Triple-vessel disease	5(10.0)
*Pre-PCI TIMI-flow grade*, *n(%)*	
0/1	46(92.0)
2/3	4(8.0)
*Post-PCI TIMI-flow grade*, *n(%)*	
0/1	0
2/3	50(100)
Rentrop grade, n(%)	0
*Medication at discharge*, *n(%)*	
ACE-i/ARBs	40(80.0)
Beta-blockers	48(96.0)
Statins	49(98.0)
Diuretics	6(12.0)
Ivabradine	3(6.0)
Ca-antagonists	2(4.0)
Anticoagulants	3(6.0)
Antithrombotic agents	49(98.0)
Nitrates	2(4.0)
*Echocardiographic data*	
LVEDVi, ml/m^2^	50±12
LVESVi, ml/m^2^	26±8
LVEF,%	49±8
N° of segments with wall motion abnormalities	7±3
TAPSE, mm	22±3
PAP, mmHg	26±8

ACE: angiotensin converting enzyme; ARB: angiotensin receptor blockade; CAD: coronary artery disease; eGFR: estimated glomerular filtration rate; GP: glycoprotein; LAD: left anterior descending artery; LVEDVi: indexed left ventricle end-diastolic volume; LVESVi: indexed left ventricle end-systolic volume; LVEF: left ventricle ejection fraction; PAP: pulmonary artery pressure; PCI: percutaneous coronary intervention; TAPSE: tricuspid annular plane systolic excursion.

**Table 2:** CMR characteristics.

VARIABLES	mean±SD
Day of exam	4±2
LVEDVi (ml/mq)	81±16
LVESVi (ml/mq)	42±13
LV EF (%)	49±9
RVEDVi (ml/mq)	64±3
RVESVi (ml/mq)	23±7
RVEF (%)	64±7
Left ventricle mass (gr/m^2^)	75±18
AWM (n° of segment)	7±3
Edema	
Mass (gr)	35[25–58]
Mass / left ventricle mass (%)	37±22/35 [21–48]
N° of segments	7±3
LGE	
Mass (g)	19[7–29]
Mass / left ventricle mass (%)	16[6–26]
N° of segments	6±4
MSI	
Value (%)	45±32
MVO	
N° of patients	36(72%)

AWM: abnormal wall motion; CMR: cardiac magnetic resonance; LGE: late gadolinium enhancement; LVEDVi: indexed left ventricle end-diastolic volume; LVESVi: indexed left ventricle end-systolic volume; LVEF: left ventricle ejection fraction; MSI: myocardial salvage index; MVO: microvascular obstruction; RVEDVi: indexed right ventricle end-diastolic volume; RVESVi: indexed right ventricle end-systolic volume; RVEF: right ventricle ejection fraction.

**Fig 1:**
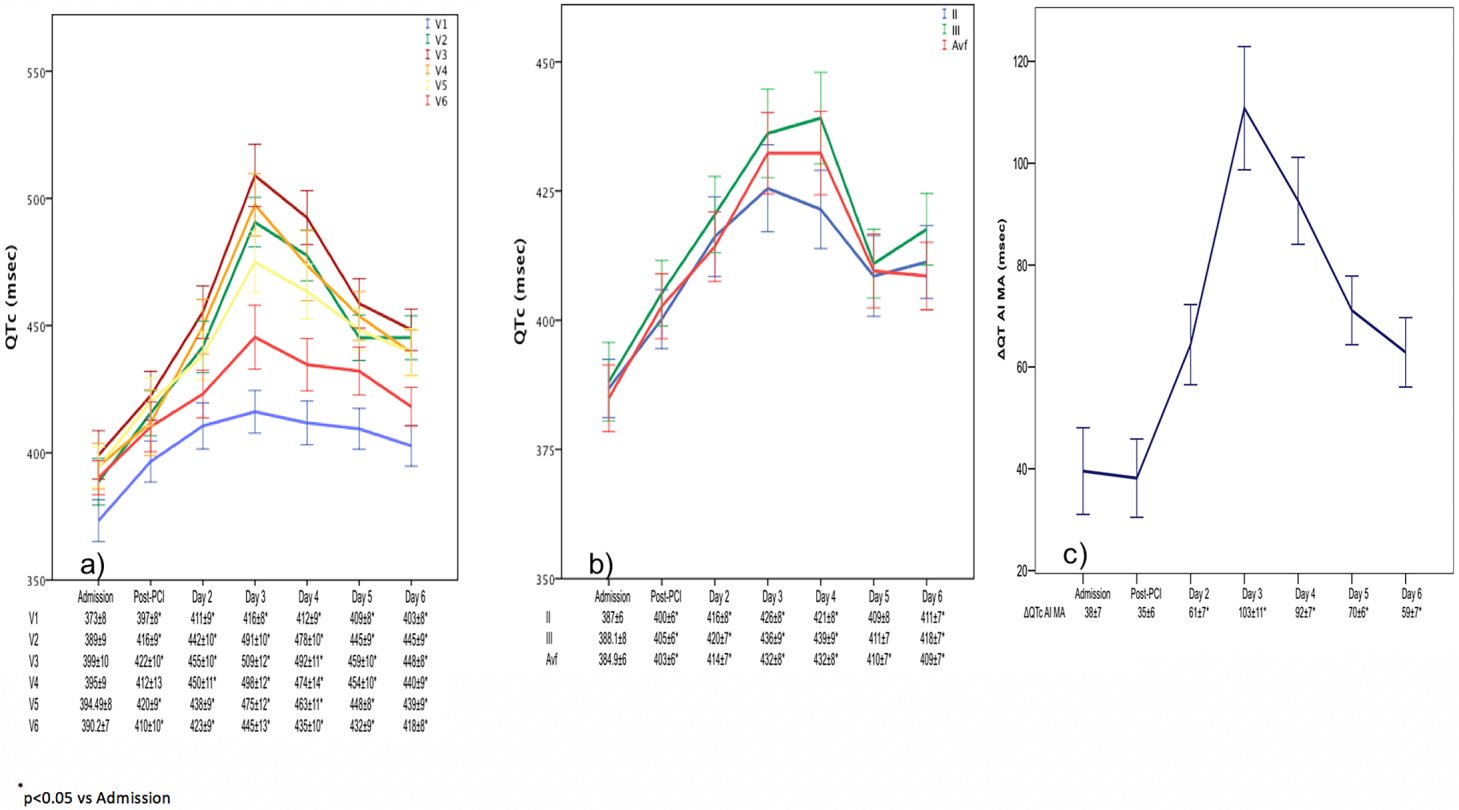
Anterior and inferior QTc derivation over time. Anterior (panel A), inferior (panel B) QTc derivations and ΔQTc-AI-MA (panel C) at different time points expressed as mean and standard error. PCI: percutaneous coronary intervention; ΔQTc-AI-MA: max anterior QTc—min inferior QTc.

Fig 1 (Panel A and B) illustrates the temporal QTc changes in all leads. The total QTc significantly increased after admission. The greatest prolongation was noted on the 3^rd^ day after STEMI (p = 0.004) and subsequently decreased through the 6^th^ day. The peak ΔQTc-AI-MA was reached on the 3^rd^ day and was significantly higher compared to that measured at admission and on the 6^th^ day (p<0.05) (Panel C).

The inter-observer agreement for QTc evaluation was 0.90 with a kappa value of 0.82 (95% confidence interval [CI] 0.60–1.0). Similarly, intra-observer agreement for the evaluation of QTc interval was 0.92 with a kappa value of 0.80 (95% CI 0.42–1.0) and 0.93 with a kappa value of 0.82 (95% CI 0.60–1.0), respectively.

**Fig 2:**
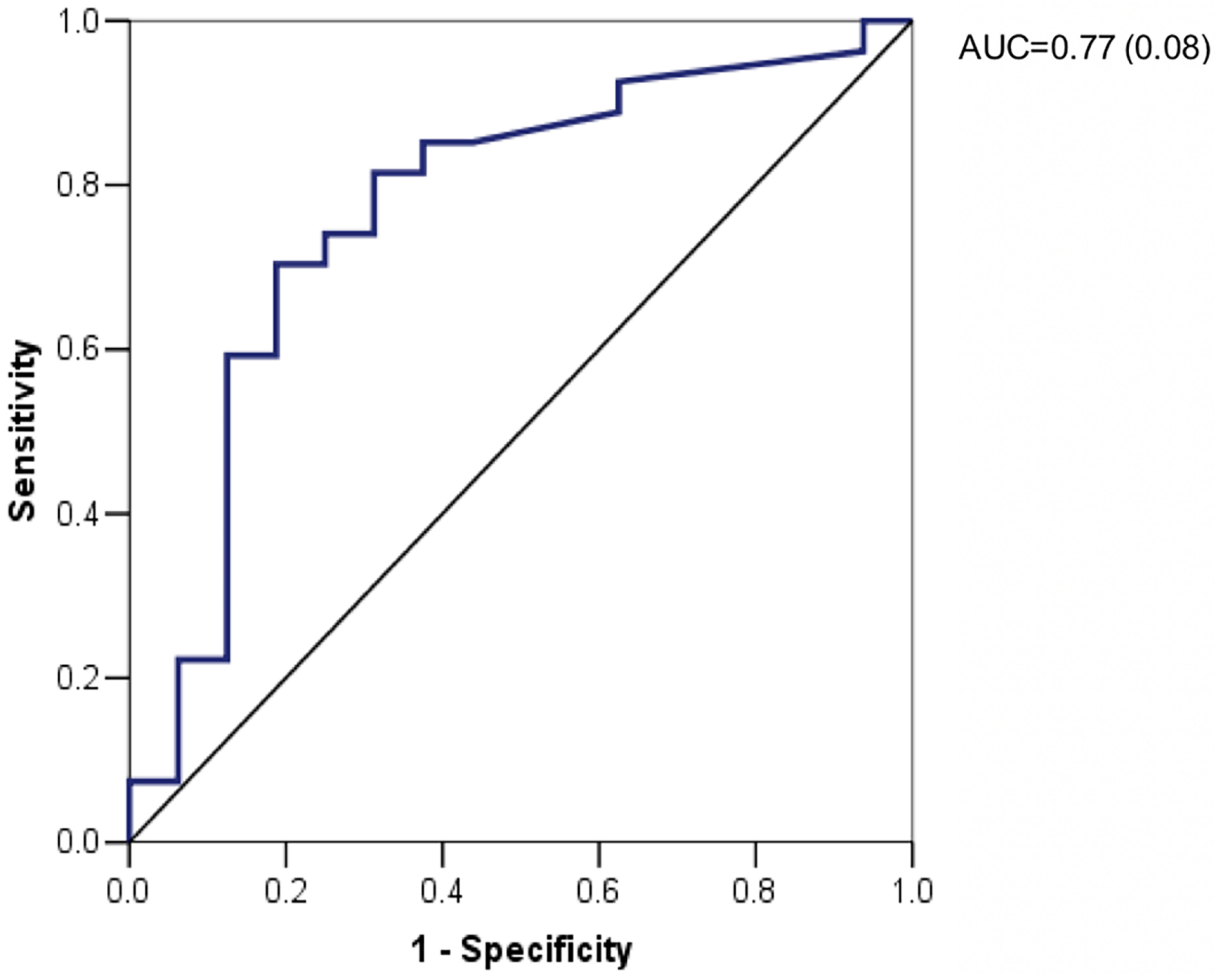
ROC curve for ΔQT AI MA at day six versus MSI <60%. ΔQTc AI MA: delta QT corrected antero-inferior max; MSI = myocardial salvage index.

ΔQTc-AI-MA demonstrated a moderate correlation with LGE mass and percentage of LGE (r = 0.50, p<0.05 and r = 0.52, p<0.05, respectively) and MSI (r = -0.42, p<0.05) (S1 Table). After performing the univariate analysis to predict LGE quantification and low MSI (S2 and S3 Tables), peak troponin I and ΔQTc-AI-MA on 6^th^ day were independent predictors of LGE expressed as absolute mass or as %LV mass on multivariate analysis (Tables 3 and 4). Moreover, on multivariate analysis (Table 5), only peak troponin I (p = 0.033) and ΔQT_C_-AI-MA on 6^th^ day (p = 0.013) remained independent predictors of MSI<0.6. The ROC curve of ΔQT_C_-AI-MA on the 6^th^day exhibited an AUC of 0.77 to predict MSI<0.6 (Fig 2) with the optimal threshold of 61msec corresponding to a sensitivity and specificity of 70% and 81%, respectively.

**Table 3:** Multivariate linear regression analysis for log-transformed LGE (gr).

	Coeff. (Std. Err.)	P-value	95% CI
Peak troponin I, ng/mL	0.004(0.001)	0.002	(0.002–0.007)
ΔQTc AI MA Day 6, msec	0.010(0.003)	0.007	(0.003–0.017)
cons	1.728(0.242)	<0.001	(1.239–2.217)
R^2^	0.420		
Adjusted R2	0.391		

LGE: late gadolinium enhancement; CI: confidence interval; ΔQTc AI MA: delta QT corrected antero-inferior max; R^2^ = coefficient of determination.

**Table 4:** Multivariate linear regression for log-transformed LGE (%).

	Coeff. (Std. Err.)	P-value	95% CI
Peak troponin I, ng/mL	0.003(0.001)	0.010	(0.008–0.006)
Number of segments with score>0	0.098(0.043)	0.029	(0.011–0.184)
ΔQTc AI MA Day 6, msec	0.008(0.003)	0.010	(0.002–0.014)
cons	1.091(0.341)	0.003	(0.399–1.782)
R^2^	0.488		
Adj R2	0.446		

LGE: late gadolinium enhancement; CI: confidence interval; ΔQTc AI MA: delta QT corrected antero-inferior max; R^2^ = coefficient of determination.

**Table 5:** Multivariate logistic regression for MSI <60%.

	OR(Srd.Err.)	P-value	95% CI
Model 1			
ΔQTc AI MA Day 6, msec	0.975(0.010)	0.015	(0.956–0.995)
LVEF, %	1.137(0.068)	0.033	(1.011–1.279)
cons	0.004(0.011)	0.066	(0–1.453)

MSI: myocardial salvage index; OR: odds ratio; CI: confidence interval; ΔQTc AI MA: delta QT corrected antero-inferior max; LGE: late gadolinium enhancement.

## Discussion

The present study found a direct relation between QT prolongation and myocardial salvage as assessed by CMR in patients with a first reperfused anterior-STEMI. In particular, we found that a significant differential value between anterior (injured myocardium) and inferior (remote myocardium) QT detected six days after pPCI correlated to and was an independent predictor of adverse MSI (<0.6) after accounting for other clinical variables. For the first time, our data provide clinical meaning of ventricular repolarization changes in reperfused anterior-STEMI applying a fast and easy tool able to stratify the prognosis of this patient subset.

CMR represents the gold standard technique for the evaluation of cardiac structure and function [12–14]. Recent data on this subset of patients have shown that CMR features indicating cardiac dysfunction, size and transmural extension of infarct, together with microvascular injury, may be more specific markers for risk assessment at the individual patient level in terms of adverse remodelling and MACE [3,15–19]. Recently, MSI, which represents the extent of irreversible myocardial damage (scar) in relation to the area supplied by the culprit vessel (AAR imaged as myocardial edema at T2-weighted sequences) has been found to have important prognostic implications for STEMI patients [20]. Masci et al. [2] showed that MSI measured within the first week after pPCI was independently related to early ST-segment resolution and adverse LV remodelling. In addition, Eitel et al. [1] demonstrated that MSI was able to predict MACE and mortality at six-month follow-up in a population of 208 post-reperfusion STEMI patients.

The most readily available test for STEMI patients at the time of presentation is the 12-lead-ECG. The ECG not only provides rationale for triage of patients with STEMI to coronary revascularization, but can also offer information on the presence and location of at-risk myocardium and MI. It is known that in the course of ischemia, lengthening of the QT is frequently observed, especially in the case of early transmural ischemia. When compared with clinically accepted indices of transmural ischemia (i.e., ST elevation [≥1 mm]) QT prolongation is the earliest ECG abnormality [4]. In the setting of STEMI, after an initial gradual increase, QTc-interval prolongation has a typical parabolic trend with the maximum length reached after 48-72hrs [21]. Importantly, the absence of QTc recovery rather than the absolute QTc following revascularization of the infarct-related vessel correlates to the peak of myocardial necrosis enzymes and to worse prognosis [22]. QT prolongation has also been considered a poor prognostic sign leading to ventricular tachycardia and/or SCD [23].

**Fig 3:**
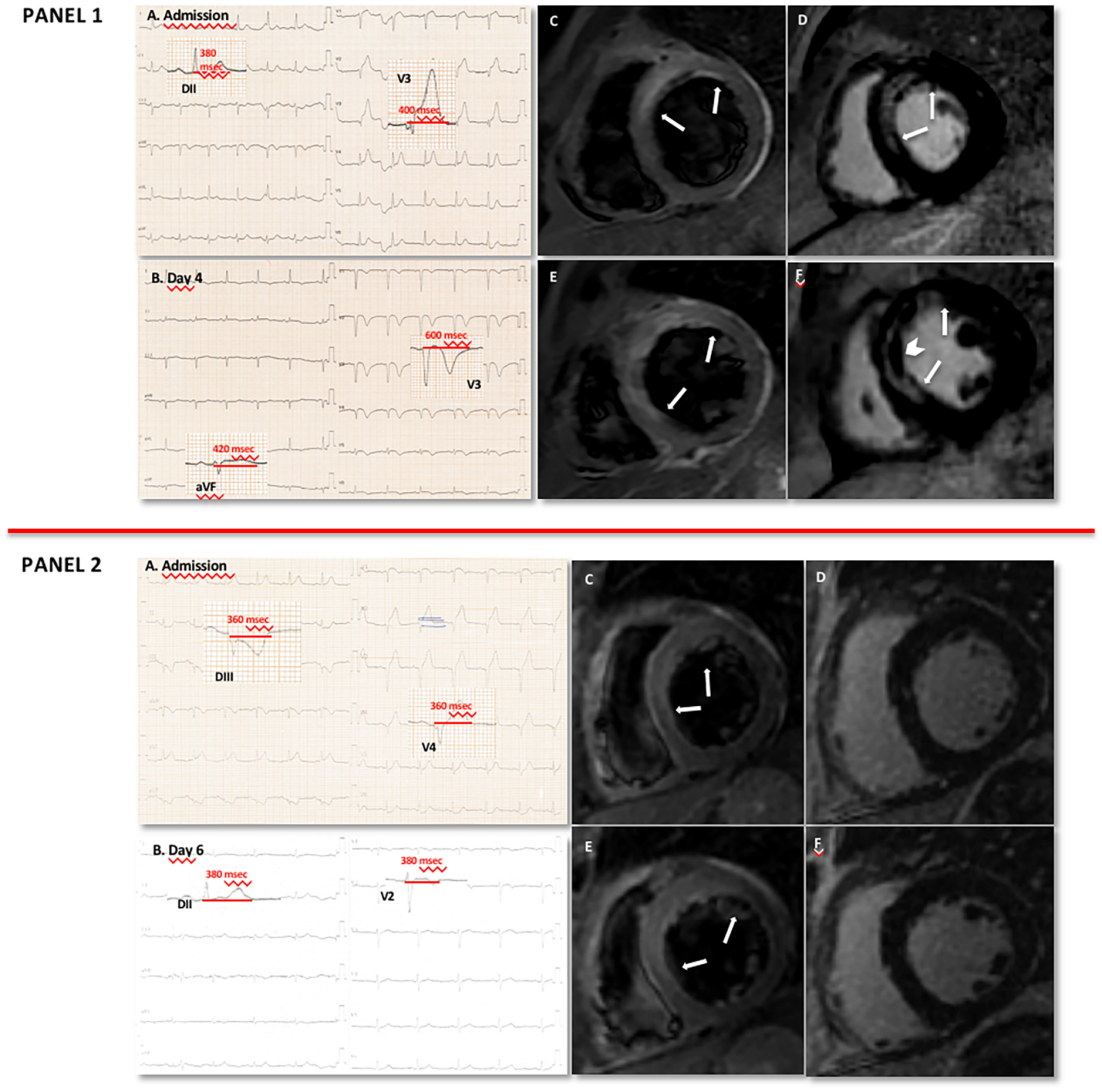
Clinical cases. **Panel 1**. A: ECG at admission. B: ECG at 6^th^ day. C, E: T2-weighted images of basal and mid-short axis views, respectively. The increased myocardial signal intensity (arrows) indicates increased water content, hence tissue edema in the anterior, antero-septal and infero-septal walls. D, F: late gadolinium enhancement (LGE) images (basal and mid-short axis views respectively). Necrosis (arrows) and microvascular obstruction (arrowhead) are shown. The edematous myocardial content was 62gr, corresponding to 56% of total left ventricular mass, and LGE was 59gr, corresponding to 54% of total left ventricular mass. The myocardial salvage index (MSI) was 0.05. ΔQTc-AI-MA (max anterior QTc—min inferior QTc) was 200msec. **Panel 2**. A: ECG at admission. B: ECG at 6^th^ day. C, E: T2-weighted images of basal and mid-short axis views, respectively. The increased myocardial signal intensity (arrows) indicates tissue edema in the anterior and antero-septal walls. D, F: LGE images (basal and mid-short axis views, respectively). The edematous myocardial content was 30gr, corresponding to 25% of total left ventricular mass. No LGE was evident. The MSI was 1. No ΔQTc-AI-MA (max anterior QTc—min inferior QTc) was present.

For the first time, we report a significant correlation between ΔQT_C_-interval prolongation at the site of injured myocardium (anterior leads) versus remote myocardium (inferior leads) and the extent of myocardial involvement assessed with CMR in patients undergoing successful pPCI for anterior STEMI. ΔQT_C_-AI-MA at 6^th^ day was inversely related to MSI and directly related to LGE extent (Fig 3). At multivariate analysis, ΔQTc-AI-MA at 6^th^ day and the peak troponin I remained independent predictors of MSI<0.6 and LGE extent. The lack of QTc-prolongation recovery at the infarction site during in-hospital monitoring indicates an at-risk patient with potential poor prognosis.

The prediction of functionally recovered myocardium in the era of pPCI is of great clinical importance in early risk stratification of STEMI patients. ECG represents a reliable and inexpensive tool in clinical practice and may serve as a surrogate of histological findings in order to reach this goal. As CMR is not widely available, these findings could be of great clinical utility. A simple tool such as the lack of QTc-prolongation recovery at the site of infarction could be a practical metric to identify a subset of patients to be addressed by CMR for enhanced prognostic stratification.

## Limitations

The following limitations should be considered. First, a relatively small population represents the sample of our study. Second, the highly selected study population may not be representative of all anterior STEMI patients. Moreover, the retrospective nature of the analysis may have influenced electrocardiogram acquisition and magnetic resonance imaging timing. On the other hand, the patients have been consecutively selected in order to avoid inclusion bias. The application of inclusion and exclusion criteria have generated a model of population very clearly defined from a pathophysiological standpoint. In fact, the aim of the study was to evaluate a specific subset of STEMI survivors, represented by patients with an apparent good prognosis (early and complete revascularization), in order to select who could benefit from a closer follow-up despite having a major myocardial tissue impairment. Third, the study does not include a comprehensive ECG correlation evaluating just QT measurements with MSI. Notably, although ST abnormalities are the best electrocardiographic parameters in acute coronary syndrome, the study design included only patients with prompt ST-elevation resolution post-PCI. Therefore, QT-interval was the only ECG parameter included in multivariate analysis. Fourth, QT interval was measured manually. However, the clinical experience of well-trained and blinded operators may be better than automated programs of calculation to measure QT interval accurately. Fifth, in the current era of pPCI for STEMI, hospital length of stay may not reach 7 days. Therefore, it would have been useful to provide a straightforward value at 3-day follow-up (peak) but the relative analysis of ΔQTC-AI-MA did not show a significant correlation with MSI. Attempting to explain this phenomenon, we postulate that during the first few days after STEMI all patients have myocardial alteration and consequently a long QT interval. Perhaps this is why 3-day follow-up may represent too short of a time interval.

## Conclusions

In a specific subset of patients with a first occurrence of anterior STEMI, early and effectively revascularized, ΔQTc is inversely correlated with CMR-derived MSI. Additional studies are needed to further validate the use and cost-effectiveness of QT_C_ changes at ECG as a gatekeeper for CMR in anterior-STEMI patients.

## Supporting information

S1 Table Correlation between cardiac magnetic resonance findings and ΔQTc AI MA (max anterior—min inferior). ΔQTc AI MA: delta QT corrected antero-inferior max; LGE: late gadolinium enhancement; MSI: myocardial salvage index; Post-PCI: post-percutaneous coronary intervention *P-value<0.05.
(DOCX)

S2 Table Univariate linear regression for log-transformed LGE (gr) and LGE(%). ACE: angiotensin converting enzyme; AWM: abnormal wall motion; CAD: coronary artery disease; CMR: cardiac magnetic resonance; GFR: glomerular filtration rate; LAD: left anterior descending artery; LVEDV: left ventricle end diastolic volume; LVEF: left ventricle ejection fraction; LVESV: left ventricle and systolic volume; PAP: pulmonary artery pressure; PCI: percutaneous coronary intervention; TTE: transthoracic echocardiography; TIMI: thrombolysis in myocardial infarction; ΔQTc AI MA = delta QT corrected antero-inferior max; ΔQTc AI ME = delta QT corrected antero-inferior mean.
(DOCX)

S3 Table Univariate logistic regression for MSI <60%. ACE: angiotensin converting enzyme; AWM: abnormal wall motion; CAD: coronary artery disease; CMR: cardiac magnetic resonance; GFR: glomerular filtration rate; LAD: left anterior descending artery; LVEDVi: indexed left ventricle end diastolic volume; LVEF: left ventricle ejection fraction; LVESVi: indexed left ventricle and systolic volume; PAP: pulmonary artery pressure; PCI: percutaneous coronary intervention; TTE: transthoracic echocardiography; TIMI: thrombolysis in myocardial infarction; ΔQTc AI MA = delta QT corrected antero-inferior max; ΔQTc AI ME = delta QT corrected antero-inferior mean.
(DOCX)

## Abbreviations

CMR: cardiac magnetic resonance
LGE: late gadolinium enhancement
LVEF: left ventricular ejection function
MI: myocardial infarction
MSI: myocardial salvage index
pPCI: primary percutaneous coronary intervention
QTc: corrected QT interval
STEMI: ST-segment elevation myocardial infarction
