# Supplementary material for: QT-interval evaluation in primary percutaneous coronary intervention of ST-segment elevation myocardial infarction for prediction of myocardial salvage index
Source: PLoS One. 2018 Feb 8;13(2):e0192220. doi: 10.1371/journal.pone.0192220 (PMC5805298; doi:10.1371/journal.pone.0192220)
Supplement: S1 Table — ΔQTc AI MA: delta QT corrected antero-inferior max; LGE: late gadolinium enhancement; MSI: myocardial salvage index; Post-PCI: post-percutaneous coronary intervention *P-value<0.05. (DOCX) [file pone.0192220.s001.docx]

**Supporting information**

**S1 Table. Correlation between cardiac magnetic resonance findings and ΔQTc AI MA (max anterior – min inferior)**

| ΔQTc AI MA  (max anter - min infer) | Edema (gr) | Edema (%) | LGE (gr) | LGE (%) | MSI |
| --- | --- | --- | --- | --- | --- |
| Admission | -0.021 | -0.010 | 0.118 | 0.09 | -0.009 |
| Post-PCI | 0.062 | 0.024 | 0.176 | 0.153 | -0.170 |
| Day 2 | 0.141 | 0.210 | 0.147 | 0.285* | 0.097 |
| Day 3 | 0.211 | 0.245 | 0.085 | 0.108 | 0.211 |
| Day 4 | 0.264 | 0.127 | 0.278 | 0.233 | -0.122 |
| Day 5 | 0.225 | 0.164 | 0.164 | 0.180 | -0.045 |
| Day 6 | 0.222 | 0.103 | 0.499* | 0.518* | -0.422* |

ΔQTc AI MA: delta QT corrected antero-inferior max; LGE: late gadolinium enhancement; MSI: myocardial salvage index; Post-PCI: post-percutaneous coronary intervention *P-value<0.05
