# Supplementary material for: QT-interval evaluation in primary percutaneous coronary intervention of ST-segment elevation myocardial infarction for prediction of myocardial salvage index
Source: PLoS One. 2018 Feb 8;13(2):e0192220. doi: 10.1371/journal.pone.0192220 (PMC5805298; doi:10.1371/journal.pone.0192220)
Supplement: S2 Table — ACE: angiotensin converting enzyme; AWM: abnormal wall motion; CAD: coronary artery disease; CMR: cardiac magnetic resonance; GFR: glomerular filtration rate; LAD: left anterior descending artery; LVEDV: left ventricle end diastolic volume; LVEF: left ventricle ejection fraction; LVESV: left ventricle and systolic volume; PAP: pulmonary artery pressure; PCI: percutaneous coronary intervention; TTE: transthoracic echocardiography; TIMI: thrombolysis in myocardial infarction; ΔQTc AI MA = delta QT corrected antero-inferior max; ΔQTc AI ME = delta QT corrected antero-inferior mean. (DOCX) [file pone.0192220.s002.docx]

**Supporting information**

**S2 Table. Univariate linear regression for log-transformed LGE (gr) and LGE(%)**

|  | LGE (gr) | | | | | LGE (%) | | | | |
| --- | --- | --- | --- | --- | --- | --- | --- | --- | --- | --- |
|  | Coef.(Srd.Err.) | | P-value | | 95% CI | Coef.(Srd.Err.) | | P-value | | 95% CI |
| Age, years | 0(0.017) | 0.988 | | (-0.035-0.034) | | -0.001(0.016) | 0.973 | | (-0.033-0.032) | |
| Female | -0.733(0.496) | 0.146 | | (-1.730-0.265) | | -0.357(0.472) | 0.453 | | (-1.305-0.591) | |
| BMI, Kg/m^2^ | 0.047(0.060) | 0.432 | | (-0.073-0.167) | | -0.011(0.056) | 0.852 | | (-0.123-0.102) | |
| Hypertension | 0.307(0.337) | 0.367 | | (-0.370-0.984) | | 0.170(0.317) | 0.593 | | (-0.467-0.807) | |
| Diabetes | 0.591(0.602) | 0.331 | | (-0.619-1.800) | | 0.550(0.563) | 0.333 | | (-0.581-1.682) | |
| Hypercholesterolemia | 0.133(0.333) | 0.693 | | (-0.538-0.803) | | 0.296(0.309) | 0.343 | | (-0.326-0.919) | |
| Family History CAD | 0.012(0.332) | 0.970 | | (-0.655-0.68) | | 0.192(0.309) | 0.537 | | (-0.430-0.814) | |
| Current smoker | 0.358(0.33) | 0.283 | | (-0.305-1.021) | | 0.380(0.308) | 0.223 | | (-0.239-0.998) | |
| Peak troponin I, ng/dL | **0.006(0.001)** | **<0.001** | | **(0.003-0.009)** | | **0.006(0.001)** | **<0.001** | | **(0.003-0.008)** | |
| Creatinine admission, mg/dl | 1.070(0.725) | 0.146 | | (-0.388-2.528) | | 0.622(0.686) | 0.370 | | (-0.759-2.002) | |
| eGFR admission, ml/min/1.73mq | -0.006(0.007) | 0.395 | | (-0.019-0.008) | | -0.002(0.006) | 0.730 | | (-0.015-0.011) | |
| Kalemia admission, mEq/l | -0.191(0.376) | 0.613 | | (-0.947-0.564) | | -0.169(0.351) | 0.632 | | (-0.875-0.537) | |
| Peak of kalemia, mEq/l | 0.617(0.473) | 0.199 | | (-0.336-1.569) | | 0.577(0.440) | 0.197 | | (-0.310-1.463) | |
| Beta-blockers | 0.365(0.605) | 0.549 | | (-0.852-1.582) | | 0.556(0.563) | 0.328 | | (-0.575-1.687) | |
| ACE-i | 0.289(0.373) | 0.442 | | (-0.461-1.04) | | 0.061(0.351) | 0.864 | | (-0.646-0.767) | |
| Diuretics | 0.907(0.682) | 0.190 | | (-0.463-2.277) | | 1.049(0.631) | 0.103 | | (-0.22-2.318) | |
| Ca-antagonist | -0.684(0.687) | 0.324 | | (-2.066-0.697) | | -0.778(0.639) | 0.230 | | (-2.064-0.507) | |
| Antithrombotic agents | -0.566(0.689) | 0.416 | | (-1.952-0.82) | | -0.653(0.642) | 0.314 | | (-1.945-0.638) | |
| Tricagrelor | 0.029(0.376) | 0.939 | | (-0.726-0.784) | | 0.099(0.351) | 0.779 | | (-0.607-0.805) | |
| Prasugrel | -0.004(0.340) | 0.990 | | (-0.687-0.678) | | 0.115(0.317) | 0.718 | | (-0.523-0.753) | |
| Clopidogrel | 0.146(0.475) | 0.760 | | (-0.808-1.100) | | -0.157(0.444) | 0.726 | | (-1.049-0.736) | |
| Statins | 0.478(0.691) | 0.492 | | (-0.910-1.867) | | 0.485(0.645) | 0.456 | | (-0.813-1.783) | |
| Aspirin | -0.832(0.832) | 0.322 | | (-2.506-0.841) | | -1.098(0.771) | 0.161 | | (-2.647-0.451) | |
| Gp IIb/IIIa inhibitors | 0.343(0.426) | 0.425 | | (-0.514-1.200) | | 0.373(0.398) | 0.354 | | (-0.427-1.172) | |
| Time-to-PCI, min | 0(0.002) | 0.939 | | (-0.003-0.004) | | 0(0.002) | 0.815 | | (-0.003-0.004) | |
| Door-to- balloon time, min | -0.007(0.005) | 0.113 | | (-0.017-0.002) | | -0.006(0.004) | 0.165 | | (-0.015-0.003) | |
| Culprit lesion proximal LAD | 0.045(0.330) | 0.891 | | (-0.618-0.708) | | 0.110(0.308) | 0.724 | | (-0.51-0.729) | |
| Double vs. single-vessel disease | 0.371(0.375) | 0.328 | | (-0.384-1.125) | | 0.473(0.347) | 0.180 | | (-0.226-1.172) | |
| Triple vs. single-vessel disease | -0.028(0.561) | 0.960 | | (-1.157-1.101) | | -0.046(0.520) | 0.930 | | (-1.092-1.000) | |
| TIMI Flow pre-PCI 0/1 | 0.206(0.607) | 0.736 | | (-1.014-1.426) | | 0.119(0.568) | 0.835 | | (-1.023-1.261) | |
| LVEDV_TTE_, ml/m^2^ | 0.010(0.014) | 0.480 | | (-0.018-0.038) | | 0(0.013) | 0.978 | | (-0.026-0.027) | |
| LVESV_TTE_, ml/m^2^ | 0.040(0.021) | 0.056 | | (-0.001-0.082) | | 0.030(0.020) | 0.131 | | (-0.009-0.07) | |
| LVEF_TTE_, % | **-0.072(0.019)** | **0.001** | | **(-0.111--0.033)** | | **-0.072(0.018)** | **<0.001** | | **(-0.107--0.036)** | |
| Number of segments with AWM | **0.148(0.046)** | **0.003** | | **(0.055-0.241)** | | **0.170(0.041)** | **<0.001** | | **(0.087-0.253)** | |
| TAPSE, mm | -0.028(0.054) | 0.610 | | (-0.138-0.082) | | -0.032(0.051) | 0.539 | | (-0.136-0.072) | |
| PAP, mmHg | 0.029(0.023) | 0.213 | | (-0.018-0.076) | | 0.021(0.022) | 0.347 | | (-0.024-0.066) | |
| LVEDV_CMR_, mL/m^2^ | **0.026(0.010)** | **0.010** | | **(0.006-0.045)** | | 0.018(0.009) | 0.064 | | (-0.001-0.036) | |
| LVESV_CMR_, mL/m^2^ | **0.045(0.011)** | **<0.001** | | **(0.022-0.067)** | | **0.036(0.011)** | **0.002** | | **(0.014-0.058)** | |
| LVEF_CMR_, % | **-0.058(0.017)** | **0.001** | | **(-0.092--0.025)** | | **-0.051(0.016)** | **0.002** | | **(-0.083--0.019)** | |
| Left ventricular mass, gr/m^2^ | -0.001(0.009) | 0.953 | | (-0.019-0.018) | | -0.007(0.009) | 0.429 | | (-0.024-0.010) | |
| ΔQTc AI ME admission, msec | 0(0.004) | 0.995 | | (-0.009-0.009) | | -0.001(0.004) | 0.807 | | (-0.009-0.007) | |
| ΔQTc AI ME Post-PCI, msec | 0.002(0.004) | 0.621 | | (-0.006-0.011) | | 0.002(0.004) | 0.621 | | (-0.006-0.010) | |
| ΔQTc AI ME Day 2, msec | 0.005(0.004) | 0.286 | | (-0.004-0.013) | | 0.007(0.004) | 0.100 | | (-0.001-0.015) | |
| ΔQTc AI ME Day 3, msec | 0.003(0.003) | 0.214 | | (-0.002-0.008) | | 0.003(0.002) | 0.215 | | (-0.002-0.008) | |
| ΔQTc AI ME Day 4, msec | **0.008(0.004)** | **0.041** | | **(0-0.015)** | | 0.006(0.003) | 0.105 | | (-0.001-0.013) | |
| ΔQTc AI ME Day 5, msec | 0.008(0.005) | 0.103 | | (-0.002-0.018) | | 0.005(0.005) | 0.266 | | (-0.004-0.014) | |
| ΔQTc AI ME Day 6, msec | **0.014(0.004)** | **0.001** | | **(0.006-0.023)** | | 0.011(0.004) | 0.006 | | (0.003-0.019) | |
| ΔQTc AI MA admission, msec | 0.003(0.003) | 0.373 | | (-0.004-0.010) | | 0.003(0.003) | 0.404 | | (-0.004-0.009) | |
| ΔQTc AI MA Post-PCI, msec | 0.005(0.004) | 0.197 | | (-0.003-0.013) | | 0.004(0.004) | 0.292 | | (-0.003-0.011) | |
| ΔQTc AI MA Day 2, msec | 0.004(0.004) | 0.330 | | (-0.004-0.011) | | 0.005(0.003) | 0.118 | | (-0.001-0.012) | |
| ΔQTc AI MA Day 3, msec | 0.003(0.002) | 0.230 | | (-0.002-0.007) | | 0.003(0.002) | 0.191 | | (-0.001-0.007) | |
| ΔQTc AI MA Day 4, msec | 0.006(0.003) | 0.065 | | (0-0.013) | | 0.005(0.003) | 0.118 | | (-0.001-0.011) | |
| ΔQTc AI MA Day 5, msec | 0.004(0.004) | 0.276 | | (-0.004-0.013) | | 0.004(0.004) | 0.316 | | (-0.004-0.011) | |
| ΔQTc AI MA Day 6, msec | **0.013(0.004)** | **0.001** | | **(0.006-0.020)** | | **0.012(0.003)** | **0.001** | | **(0.005-0.019)** | |

ACE: angiotensin converting enzyme; AWM: abnormal wall motion; CAD: coronary artery disease; CMR: cardiac magnetic resonance; GFR: glomerular filtration rate; LAD: left anterior descending artery; LVEDV: left ventricle end diastolic volume; LVEF: left ventricle ejection fraction; LVESV: left ventricle and systolic volume; PAP: pulmonary artery pressure; PCI: percutaneous coronary intervention; TTE: transthoracic echocardiography; TIMI: thrombolysis in myocardial infarction; ΔQTc AI MA= delta QT corrected antero-inferior max; ΔQTc AI ME= delta QT corrected antero-inferior mean.
