# Supplementary material for: QT-interval evaluation in primary percutaneous coronary intervention of ST-segment elevation myocardial infarction for prediction of myocardial salvage index
Source: PLoS One. 2018 Feb 8;13(2):e0192220. doi: 10.1371/journal.pone.0192220 (PMC5805298; doi:10.1371/journal.pone.0192220)
Supplement: S3 Table — ACE: angiotensin converting enzyme; AWM: abnormal wall motion; CAD: coronary artery disease; CMR: cardiac magnetic resonance; GFR: glomerular filtration rate; LAD: left anterior descending artery; LVEDVi: indexed left ventricle end diastolic volume; LVEF: left ventricle ejection fraction; LVESVi: indexed left ventricle and systolic volume; PAP: pulmonary artery pressure; PCI: percutaneous coronary intervention; TTE: transthoracic echocardiography; TIMI: thrombolysis in myocardial infarction; ΔQTc AI MA = delta QT corrected antero-inferior max; ΔQTc AI ME = delta QT corrected antero-inferior mean. (DOCX) [file pone.0192220.s003.docx]

**Supporting information**

**S3 Table. Univariate logistic regression for MSI <60%**

|  | OR(Srd.Err.) | | P-value | | 95% CI |
| --- | --- | --- | --- | --- | --- |
| Age, years | 1.019(0.032) | 0.540 | | (0.959-1.084) | |
| **Female** | **11.923(13.642)** | **0.030** | | **(1.266-112.287)** | |
| **BMI, Kg/m^2^** | **0.788(0.096)** | **0.050** | | **(0.621-1.000)** | |
| Hypertension | 0.324(0.216) | 0.092 | | (0.087-1.200) | |
| Diabetes | - | - | | - | |
| Hypercholesterolemia | 0.567(0.347) | 0.354 | | (0.171-1.883) | |
| Family history of CAD | 2.083(1.247) | 0.220 | | (0.644-6.734) | |
| Current smoker | 0.419(0.253) | 0.149 | | (0.129-1.366) | |
| **Peak troponin I, ng/mL** | **0.979(0.008)** | **0.012** | | **(0.964-0.995)** | |
| Admission creatinine, mg/dl | 0.063(0.101) | 0.085 | | (0.003-1.458) | |
| Admission eGFR, ml/min/1.73mq | 1.012(0.013) | 0.325 | | (0.988-1.037) | |
| Kalemia admission, mEq/l | 2.182(1.492) | 0.254 | | (0.571-8.331) | |
| Highest kalemia, mEq/l | 0.458(0.405) | 0.378 | | (0.081-2.593) | |
| Beta-blockers | 0.569(0.679) | 0.636 | | (0.055-5.909) | |
| **ACE-i** | **0.098(0.107)** | **0.033** | | **(0.012-0.833)** | |
| Diuretics | - | - | | - | |
| Ca-antagonist | 0.882(1.113) | 0.921 | | (0.074-10.464) | |
| Anticoagulant agents | - | - | | - | |
| Tricagrelor | 1.786(1.175) | 0.378 | | (0.492-6.484) | |
| Prasugrel | 0.943(0.571) | 0.923 | | (0.288-3.092) | |
| Clopidogrel | 0.255(0.287) | 0.224 | | (0.028-2.309) | |
| Statins | 0.882(1.113) | 0.921 | | (0.074-10.464) | |
| Aspirin | - | - | | - | |
| Gp IIb/IIIa inhibitors | 0.867(0.674) | 0.854 | | (0.189-3.981) | |
| Time-to-PCI, min | 0.996(0.003) | 0.261 | | (0.989-1.003) | |
| Door-to-balloon time, min | 0.996(0.003) | 0.261 | | (0.989-1.003) | |
| Culprit lesion proximal LAD | 0.882(0.52) | 0.832 | | (0.278-2.803) | |
| Double vs. single-vessel disease | 1.010(0.679) | 0.988 | | (0.270-3.773) | |
| Triple vs. single-vessel disease | 1.212(1.196) | 0.845 | | (0.175-8.389) | |
| TIMI Flow pre-PCI 0/1 | 0.533(0.558) | 0.548 | | (0.069-4.150) | |
| LVEDVi_TTE_, ml/m^2^ | 0.959(0.027) | 0.132 | | (0.908-1.013) | |
| **LVESVi_TTE_, ml/m^2^** | **0.905(0.043)** | **0.033** | | **(0.825-0.992)** | |
| **LVEF_TTE_, %** | **1.127(0.054)** | **0.013** | | **(1.025-1.239)** | |
| Number of segments with score>0 | 1.023(0.094) | 0.800 | | (0.855-1.225) | |
| TAPSE, mm | 1.024(0.104) | 0.817 | | (0.839-1.250) | |
| PAP, mmHg | 1.000(0.042) | 0.994 | | (0.921-1.087) | |
| **LVESVi_CMR_, mL/m^2^** | **0.947(0.022)** | **0.020** | | **(0.905-0.992)** | |
| **LVEDVi_CMR_, mL/m^2^** | **0.897(0.032)** | **0.002** | | **(0.836-0.962)** | |
| **LVEF_CMR_, %** | **1.095(0.043)** | **0.020** | | **(1.014-1.183)** | |
| Left ventricular mass, gr/m^2^ | 0.993(0.017) | 0.657 | | (0.961-1.026) | |
| ΔQTc AI ME admission, msec | 0.994(0.008) | 0.468 | | (0.978-1.010) | |
| ΔQTc AI ME Post-PCI, msec | 0.991(0.009) | 0.318 | | (0.974-1.009) | |
| ΔQTc AI ME Day 2, msec | 0.998(0.008) | 0.839 | | (0.983-1.014) | |
| ΔQTc AI ME Day 3, msec | 1.004(0.005) | 0.360 | | (0.995-1.014) | |
| **ΔQTc AI ME Day 4, msec** | **0.982(0.008)** | **0.033** | | **(0.965-0.999)** | |
| **ΔQTc AI ME Day 5, msec** | **0.972(0.011)** | **0.013** | | **(0.951-0.994)** | |
| **ΔQTc AI ME Day 6, msec** | **0.969(0.011)** | **0.006** | | **(0.947-0.991)** | |
| ΔQTc AI MA admission, msec | 0.995(0.006) | 0.474 | | (0.983-1.008) | |
| ΔQTc AI MA Post-PCI, msec | 0.986(0.009) | 0.123 | | (0.968-1.004) | |
| ΔQTc AI MA Day 2, msec | 0.999(0.007) | 0.936 | | (0.987-1.013) | |
| ΔQTc AI MA Day 3, msec | 1.005(0.004) | 0.212 | | (0.997-1.014) | |
| ΔQTc AI MA Day 4, msec | 0.992(0.006) | 0.201 | | (0.979-1.004) | |
| ΔQTc AI MA Day 5, msec | 0.997(0.008) | 0.649 | | (0.982-1.012) | |
| **ΔQTc AI MA Day 6, msec** | **0.974(0.009)** | **0.005** | | **(0.956-0.992)** | |

ACE: angiotensin converting enzyme; AWM: abnormal wall motion; CAD: coronary artery disease; CMR: cardiac magnetic resonance; GFR: glomerular filtration rate; LAD: left anterior descending artery; LVEDVi: indexed left ventricle end diastolic volume; LVEF: left ventricle ejection fraction; LVESVi: indexed left ventricle and systolic volume; PAP: pulmonary artery pressure; PCI: percutaneous coronary intervention; TTE: transthoracic echocardiography; TIMI: thrombolysis in myocardial infarction; ΔQTc AI MA= delta QT corrected antero-inferior max; ΔQTc AI ME= delta QT corrected antero-inferior mean.
